# A retrospective analysis of the prognostic implications of glycemic variability on all-cause mortality in critically ill patients with mitral valve disease

**DOI:** 10.3389/fendo.2025.1620762

**Published:** 2025-07-14

**Authors:** Jie Peng, Xingzhan Zhang, Yunjing Mai, Hongzhi Chen, Dakai Liang, Shaobo Li, Huanhuan Wu, Chao Li, YuanShuo Ge, Guangdong Wang, Ling Zhao

**Affiliations:** ^1^ Department of Intensive Care Unit, The People’s Hospital Medical Group of Xiangzhou, Zhuhai, Guangdong, China; ^2^ Department of Cardiology, People’s Hospital of Yangjiang, Yangjiang, Guangdong, China; ^3^ Department of Cardiovascular and Metabolism Medicine, The People’s Hospital Medical Group of Xiangzhou, Zhuhai, Guangdong, China; ^4^ Jinzhou Medical University, Jinzhou, Liaoning, China; ^5^ Department of Respiratory and Critical Care Medicine, First Affiliated Hospital of Xi’an Jiaotong University, Xi’an, Shanxi, China

**Keywords:** glycemic variability, mitral valve disease, MIMIC-IV database, all-cause mortality, critical ill patient

## Abstract

**Background:**

Mitral valve disease is associated with higher cardiovascular morbidity and mortality. Glycemic variability (GV), reflecting blood glucose fluctuations, acts as an independent indicator for results in critically ill patients. However, it’s effect on patients in the ICU with mitral valve disease is unclear.

**Methods:**

All blood glucose measurements of patients with mitral valve disease were extracted from MIMIC-IV database. GV was assessed using the coefficient of variation of glucose levels. Cox hazard regression models and restrictive cubic spline (RCS) were applied to examine the link between GV and outcomes. A threshold effect analysis was also conducted to explore potential inflection points. Subgroup analyses were performed to assess consistency across demographics and clinical subgroups.

**Results:**

The study of 3,378 adults with mitral valve disease found that higher GV was significantly associated with increased 28-day and 90-day mortality, as shown by Cox regression analysis. Subgroup analyses confirmed these findings.

**Conclusions:**

For ICU patients with mitral valve disease, elevated GV was an independent predictor of short-term mortality. GV was recommended for consideration in risk stratification and glucose management strategies, particularly in non-diabetic critically ill populations. Monitoring and targeting GV were proposed to new avenues for improving clinical outcomes in this high-risk group.

## Introduction

Mitral valve disease encompasses a range of conditions that impair the structure or function of the mitral valve, which directs blood flow between the left atrium and the left ventricle. The underlying etiologies are diverse and include rheumatic fever, degenerative changes, infections, ischemia-induced cardiomyopathy, and congenital anomalies (1). The burden of mitral valve disease, a primary type of heart valve disorder, was escalating globally. This disease mainly included mitral stenosis, mitral regurgitation, and mitral valve prolapse, all of which could cause heart failure and various serious cardiac complications (2). There had been a significant rise in the incidence of mitral regurgitation in recent years (3). From 2006 to 2019, the age-standardized hospitalization rate for mitral valve prolapse increased by 60% (3). Research indicated that individuals with severe mitral regurgitation experienced a higher death rate compared to those without it (33% compared to 14%) (4). Despite advances in medical and surgical treatments, mitral valve disease remained challenging to manage due to its heterogeneous etiology, asymptomatic progression in early stages (5), and frequent coexistence with comorbid conditions such as atrial fibrillation, pulmonary hypertension, and metabolic disorders (6). These factors often delayed diagnosis and complicated treatment decision-making. Studies showed that a significant elevation in endothelial microparticles was observed among individuals with mitral valve disease, potentially exacerbating mitral valve endothelial dysfunction and contributing to the progression of the disease (7). Furthermore, metabolic disturbances were identified in patients with mitral valve disease, and these alterations might have served as potential biomarkers for early diagnosis (8).

Hospitalized patients often experienced both hyperglycemia and hypoglycemia, which were associated with higher rates of complications and death, independent of diabetes status (9–11). GV was recently introduced as an additional measure for evaluating glucose control, and it was suggested to have a major effect on the pathophysiological processes associated with dysglycemia (12). The term GV described variations in blood glucose concentrations and was considered an emerging indicator of insufficient glycemic control and greater susceptibility to complications (13). Research conducted in both laboratory settings and with human participants showed that GV resulted in elevated oxidative stress and dysfunction of the endothelium, as opposed to continuous hyperglycemia (11, 14). Notably, GV correlated with a greater likelihood of experiencing major adverse cardiovascular events (MACEs), including non-fatal myocardial infarction, non-fatal stroke, and mortality related to cardiovascular disease (15).

In the context of mitral valve disease, there was an elevation in endothelial microparticles, which disrupted nitric oxide synthesis and enhanced superoxide production within mitral valve endothelial cells (16). GV was known to induce endothelial damage through acute fluctuations in blood glucose levels, which might surpassed the detrimental effects of chronic hyperglycemia (17). Growing evidence indicated a significant correlation between increased GV and heightened all-cause mortality (9, 11). However, it remained unclear whether this association was applicable to critically ill patients with mitral valve disease, who typically presented with more severe physiological conditions. These patients often exhibited hemodynamic instability, multi-organ dysfunction, and a high burden of metabolic stress, making early risk stratification particularly difficult (18).

Consequently, evaluating the potential of GV as a predictor of all-cause mortality in this patient population could have been instrumental in identifying individuals at elevated risk, thereby informing healthcare management strategies and potentially guiding timely interventions. In light of the current research landscape, the present study was conducted to investigate the role of GV in predicting all-cause mortality among critically ill patients with mitral valve disease through an analysis of the MIMIC-IV database.

## Materials

### Study population

For this retrospective analysis, health-related data were sourced from the MIMIC-IV (version 3.1) database, which was a comprehensive resource developed by the Massachusetts Institute of Technology (MIT) Laboratory for Computational Physiology in partnership with various research collaborators. The dataset comprised extensive and high-quality clinical records of patients admitted to either the emergency department or ICU at Beth Israel Deaconess Medical Center (BIDMC) during the period 2008–2022 (19). Jie Peng, an author, followed the necessary protocols for database access and was responsible for extracting the data. Strict procedures were applied during the data extraction process to guarantee accuracy and consistency. As the MIMIC-IV database is de-identified, informed consent from patients was not required.

Participants identified with mitral valve conditions were incorporated into this study according to the standards set forth in the 9th and 10th editions of the International Classification of Diseases (ICD-9 and ICD-10). The included conditions encompassed a broad range of mitral valve disorders, including rheumatic and non-rheumatic mitral stenosis or regurgitation, mixed mitral valve disease, mitral annular calcification, functional mitral insufficiency, and congenital mitral valve malformations. The specific ICD codes used for case identification are listed in **Supplementary Table S1**.

**Figure 1:**
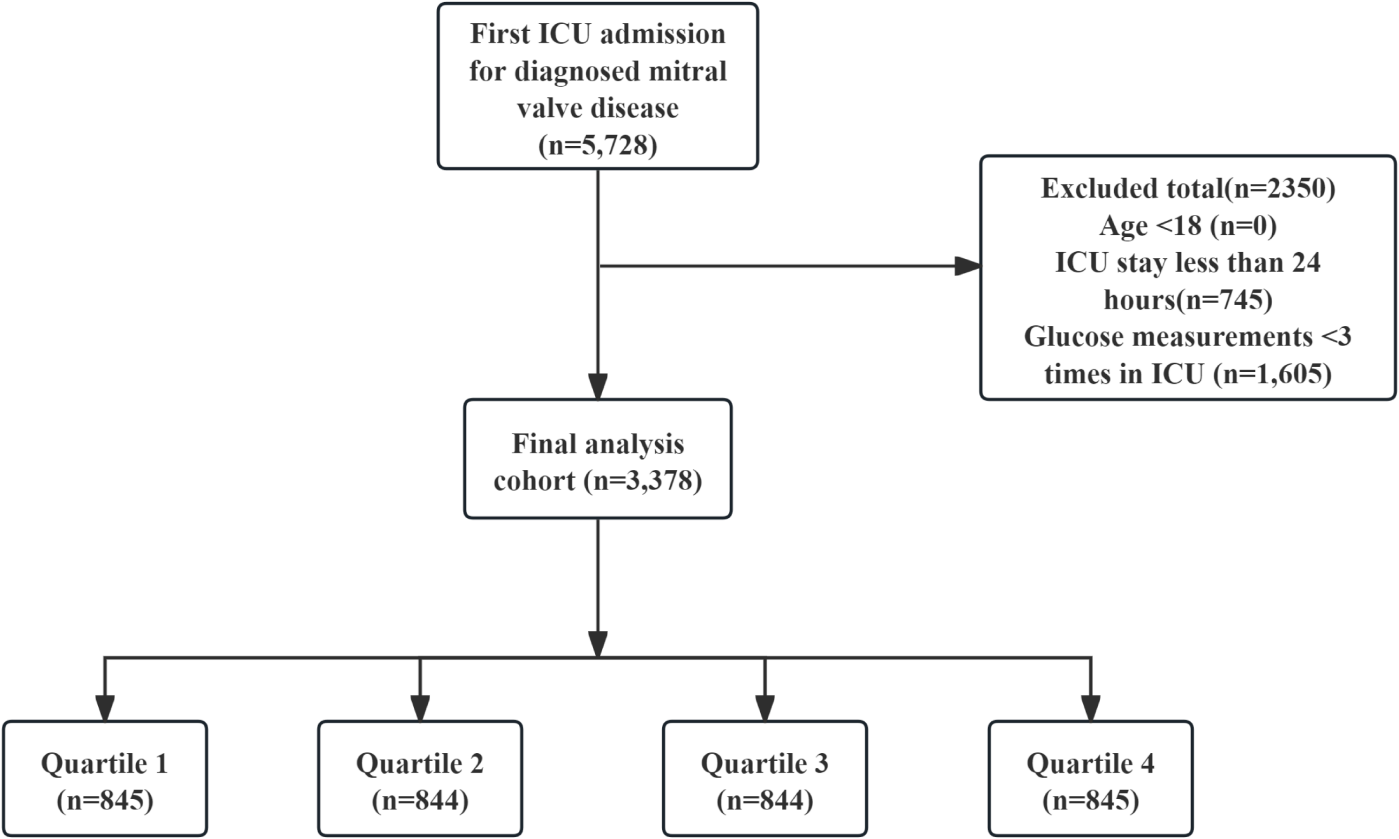
Flowchart of the study.

The following criteria led to exclusion: (1) patients who experienced multiple ICU admissions for confirmed mitral valve issues, from whom data were gathered solely from the first admission; (2) individuals younger than 18 years at the time of their initial admission; (3) patients whose ICU stay was less than 24 hours; (4) patients who had their glucose levels measured fewer than three times during their ICU admission. Eventually, the study included 3378 participants who were categorized into four groups according to the quartiles of GV (**Figure 1**).

### Data collection

Data were extracted using PostgreSQL (version 13.7.2) and Navicat Premium (version 16) by executing Structured Query Language (SQL).

The variables that might have influenced outcomes were grouped into six key categories: (1) demographics, encompassing factors like age, sex, race, and weight; (2) vital signs, which included heart rate, systolic blood pressure (SBP), diastolic blood pressure (DBP), respiratory rate, temperature, and oxygen saturation levels; (3) illness severity assessments, such as the Charlson Comorbidity Index (CCI), Sequential Organ Failure Assessment (SOFA), and the Logistic Organ Dysfunction System (LODS); (4) comorbid conditions, which consisted of congestive heart failure, chronic lung disease, hypertension, acute kidney injury, and sepsis. Congestive heart failure, chronic lung disease, and diabetes were identified via the CCI, while hypertension was extracted using ICD codes. Sepsis was defined according to the Sepsis-3.0 criteria (20), and acute kidney injury was determined using KDIGO guidelines (21); (5) urine output and various laboratory metrics, which included urine output volume, red blood cell (RBC) count, white blood cell (WBC) count, platelet levels, serum creatinine, Prothrombin time (PT), serum sodium, serum potassium, blood urea nitrogen (BUN), International normalized ratio (INR), Partial thromboplastin time (PTT), and GV; and (6) therapeutic interventions, which involved norepinephrine, neuromuscular blockers, insulin, mechanical ventilation, and continuous renal replacement therapy.

The follow-up began on the admission date and ended on the date of death. The patient’s death date was obtained from hospital and status records, with hospital records prioritized. Post-discharge deaths were confirmed by matching with social security death data (19). In this research, blood glucose readings were solely gathered throughout patients’ stays in the ICU to guarantee that GV accurately reflected changes in glucose levels during this crucial time. The coefficient of variation (CV) was employed to quantify GV, serving as a standardized measure that adjusted for variations in mean glucose levels. To calculate the CV, the standard deviation of glucose measurements was divided by the average glucose value, and this quotient was then multiplied by 100%, represented mathematically as: GV = (standard deviation of blood glucose/average blood glucose) × 100% (22). All laboratory parameters and disease severity scores were derived from data collected within the first 24 hours of ICU admission.

The missing variables and their missing proportions were presented in **Supplementary Table S2**. To reduce the likelihood of bias, any variables exhibiting over 20% of missing data were excluded from the analysis. In cases where datasets had less than 20% missing information, the missing values were imputed using a random forest algorithm through the “mice” package in R software (23, 24).

### Clinical outcomes

The main result of this study was the all-cause mortality rate at 28 days following hospital admission, while the secondary outcome was the all-cause mortality rate at 90 days post-admission to the hospital.

### Analysis of statistics

The Kolmogorov–Smirnov test was utilized to evaluate continuous variables (25). Since all continuous variables exhibited non-normal distribution, descriptive statistics were reported as the median (inter-quartile range, IQR), while the Mann–Whitney U test was employed to analyze differences across groups. Differences between groups were assessed using Pearson’s chi-squared test, and categorical variables were expressed as percentages (%). GV was categorized into four groups based on the 25th, 50th, and 75th percentiles. The Kaplan-Meier survival analysis was conducted to examine endpoint incidence rates among groups with differing GV levels, with the Log-rank test employed to assess differences. To investigate the relationship between GV and endpoints, Cox proportional hazards models were applied to calculate the hazard ratio (HR) and 95% confidence interval (CI), with specific models adjusted accordingly. Factors that could confound the results were identified based on a P-value less than 0.05 in univariate analysis, and clinically relevant variables associated with prognosis were included in the multivariate model: model 1 was unadjusted; model 2 was adjusted for sex, race, insurance, smoking status, age, and weight; model 3 included adjustments for sex, race, insurance, smoking status, congestive heart failure, diabetes, insulin, age, weight, SBP, CCI, potassium, creatinine, and INR. Additionally, the nonlinear association between the GV index and all-cause mortality at 28 days and 90 days was explored using an RCS regression model with three knots. The relationship was further examined using a threshold effect analysis to detect potential inflection points.

The models incorporated GV as both continuous and ordinal variables, utilizing the first quartile of the GV index as the reference category. Further analyses were stratified by factors including age (< 65 and ≥ 65 years), sex, presence of chronic pulmonary disease, diabetes, hypertension, sepsis, and insulin administration, in order to evaluate the GV index’s consistent prognostic value for both primary and secondary outcomes. Likelihood ratio tests were employed to assess the interactions between the GV index and the stratification factors. A two-tailed P value of less than 0.05 was considered statistically significant. Sensitivity analyses included an assessment of the association between GV and 60-day all-cause mortality as an alternative outcome. Furthermore, analyses of 28-day and 90-day mortality were repeated after excluding patients with diabetes to evaluate the robustness of the results. Statistical evaluations were conducted using R software (version 4.2.2) alongside SPSS 22.0 (IBM SPSS Statistics, Armonk, NY, USA).

## Results

**Figure 2:**
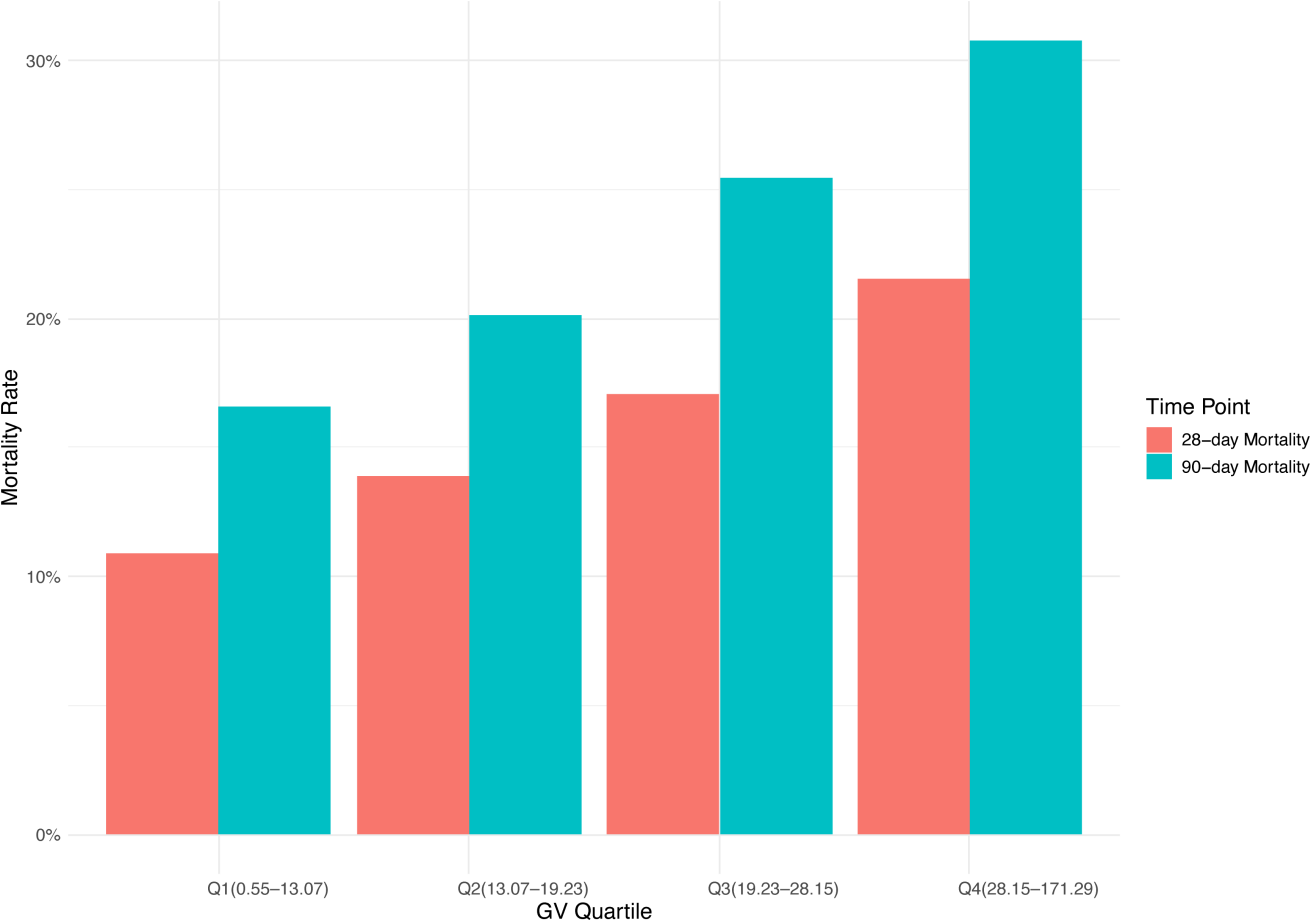
Bar graph.

This study enrolled 3,378 critically ill patients diagnosed with mitral valve disease. The median age was 74.47 years (IQR: 64.39–82.78), with males comprising 53% (n = 1,781) of the cohort. Based on GV distribution, patients were stratified into four quartiles: Q1 (0.55–13.07), Q2 (13.07–19.23), Q3 (19.23–28.15), and Q4 (28.15–171.29). The median GV among participants was 19.23 (IQR: 13.07–28.15). Hospital mortality rates were 16% at 28 days and 23% at 90 days (**Table 1**). **Figure 2** shows the association between GV and mortality across four quartiles.

**Table 1:** Baseline characteristics.

Characteristic	Overall N = 3,378	Q1 N = 845	Q2 N = 844	Q3 N = 844	Q4 N = 845	*P*-value
GV quartile range	(0.55–171.29)	(0.55–13.07)	(13.07–19.23)	(19.23–28.15)	(28.15–171.29)	
Age (year)	74.47 (64.39, 82.78)	74.26 (63.34, 83.09)	74.28 (63.58, 83.25)	75.27 (65.91, 83.18)	74.13 (65.34, 81.74)	0.287
Sex, n (%)						0.069
Female	1,597 (47%)	402 (48%)	367 (43%)	410 (49%)	418 (49%)	
Male	1,781 (53%)	443 (52%)	477 (57%)	434 (51%)	427 (51%)	
Race, n (%)						0.028
Black	272 (8%)	64 (8%)	54 (6%)	73 (9%)	81 (10%)	
Other	647 (19%)	144 (17%)	161 (19%)	158 (19%)	184 (22%)	
White	2,459 (73%)	637 (75%)	629 (75%)	613 (73%)	580 (69%)	
Weight (Kg)	76.00 (64.10, 90.90)	76.60 (64.40, 91.20)	76.70 (64.00, 92.00)	74.75 (64.70, 89.00)	75.80 (63.50, 91.20)	0.578
Smoker, n (%)	353 (10%)	73 (9%)	91 (11%)	92 (11%)	97 (11%)	0.241
Insurance, n (%)						<0.001
Medicaid	250 (7%)	59 (7%)	71 (8%)	62 (7%)	58 (7%)	
Medicare	1,900 (56%)	452 (53%)	487 (58%)	482 (57%)	479 (57%)	
Other	711 (21%)	153 (18%)	159 (19%)	199 (24%)	200 (24%)	
Private	517 (15%)	181 (21%)	127 (15%)	101 (12%)	108 (13%)	
Heart rate (bmp)	82.16 (74.29, 92.96)	81.05 (73.64, 91.96)	82.21 (74.69, 92.68)	82.00 (74.03, 92.76)	83.45 (74.43, 95.41)	0.070
SBP (mmHg)	109.63 (102.91, 119.07)	110.76 (103.43, 120.38)	109.31 (102.55, 118.54)	109.01 (102.90, 117.75)	109.93 (102.44, 118.78)	0.024
DBP (mmHg)	59.03 (52.79, 65.70)	60.65 (54.38, 66.80)	58.70 (52.77, 65.28)	58.59 (52.26, 65.00)	57.97 (51.96, 65.24)	<0.001
Respiratory rate (bmp)	19.08 (16.95, 21.81)	18.88 (16.85, 21.31)	19.09 (16.96, 21.75)	19.18 (16.94, 22.02)	19.28 (17.13, 22.07)	0.094
Temperature (°C)	36.74 (36.52, 36.99)	36.76 (36.55, 36.98)	36.74 (36.52, 37.00)	36.73 (36.51, 37.01)	36.73 (36.50, 36.98)	0.406
Spo_2_ (%)	97.20 (95.80, 98.48)	97.06 (95.77, 98.31)	97.21 (95.81, 98.43)	97.16 (95.74, 98.48)	97.46 (95.96, 98.61)	0.051
SOFA	2.00 (0.00, 4.00)	1.00 (0.00, 3.00)	2.00 (0.00, 4.00)	2.00 (1.00, 4.00)	2.00 (1.00, 4.00)	<0.001
LODS	5.00 (3.00, 7.00)	4.00 (3.00, 6.00)	5.00 (3.00, 7.00)	6.00 (4.00, 8.00)	6.00 (4.00, 8.00)	<0.001
CCI	6.00 (4.00, 8.00)	5.00 (3.00, 7.00)	6.00 (4.00, 8.00)	6.00 (5.00, 8.00)	7.00 (5.00, 9.00)	<0.001
Congestive heart failure, n (%)	2,380 (70%)	535 (63%)	580 (69%)	619 (73%)	646 (76%)	<0.001
Chronic pulmonary disease, n (%)	1,100 (33%)	266 (31%)	250 (30%)	293 (35%)	291 (34%)	0.075
Diabetes, n (%)	1,136 (34%)	153 (18%)	205 (24%)	304 (36%)	474 (56%)	<0.001
Hypertension, n (%)	2,594 (77%)	617 (73%)	636 (75%)	659 (78%)	682 (81%)	0.001
AKI, n (%)	2,948 (87%)	689 (82%)	735 (87%)	765 (91%)	759 (90%)	<0.001
Sepsis, n (%)	1,925 (57%)	403 (48%)	483 (57%)	501 (59%)	538 (64%)	<0.001
Urine output (mL)	1,482.50 (885.00, 2,395.00)	1,550.00 (975.00, 2,430.00)	1,532.50 (972.50, 2,425.00)	1,469.00 (901.00, 2,320.00)	1,360.00 (729.00, 2,310.00)	0.001
RBC (10^9^/L)	3.38 (2.99, 3.89)	3.45 (3.01, 3.97)	3.40 (3.00, 3.96)	3.37 (3.00, 3.79)	3.34 (2.95, 3.85)	0.010
WBC (10^9^/L)	11.70 (8.75, 15.30)	11.33 (8.50, 14.67)	11.80 (8.80, 15.48)	11.80 (8.78, 15.65)	11.90 (8.90, 15.80)	0.037
Platelet (10^9^/L)	170.00 (127.00, 226.33)	167.00 (127.00, 224.50)	166.61 (126.75, 215.13)	171.67 (126.00, 228.42)	175.25 (128.33, 235.33)	0.209
GV	19.23 (13.07, 28.15)	9.57 (6.74, 11.52)	16.09 (14.66, 17.69)	23.07 (20.84, 25.33)	36.94 (31.89, 45.82)	<0.001
Sodium (mmol/L)	138.00 (135.50, 140.33)	138.00 (135.67, 140.00)	138.00 (135.75, 140.33)	138.00 (135.50, 140.50)	137.60 (135.00, 140.00)	0.021
Potassium (mmol/L)	4.27 (3.93, 4.65)	4.23 (3.90, 4.57)	4.25 (3.95, 4.60)	4.27 (3.95, 4.65)	4.33 (3.95, 4.77)	0.004
BUN (mg/dL)	23.42 (16.00, 39.50)	20.00 (14.00, 33.00)	21.42 (15.67, 37.42)	24.67 (16.50, 41.25)	28.67 (18.00, 46.50)	<0.001
Creatinine (mg/dL)	1.15 (0.85, 1.78)	1.00 (0.80, 1.47)	1.10 (0.80, 1.65)	1.19 (0.85, 1.83)	1.35 (0.95, 2.20)	<0.001
INR	1.35 (1.20, 1.60)	1.33 (1.20, 1.53)	1.35 (1.20, 1.56)	1.39 (1.20, 1.63)	1.37 (1.20, 1.65)	0.014
PT (s)	14.87 (13.33, 17.45)	14.60 (13.20, 16.80)	14.80 (13.37, 16.87)	15.13 (13.40, 17.76)	14.90 (13.30, 18.00)	0.013
PTT (s)	33.60 (29.08, 44.93)	32.80 (29.00, 43.06)	33.36 (28.58, 43.86)	33.85 (29.28, 45.68)	34.47 (29.33, 47.93)	0.014
Norepinephrine, n (%)	1,050 (31%)	173 (20%)	263 (31%)	296 (35%)	318 (38%)	<0.001
Neuro-muscle blocker, n (%)	86 (3%)	5 (1%)	21 (2%)	29 (3%)	31 (4%)	<0.001
Insulin, n (%)	2,000 (59%)	420 (50%)	485 (57%)	495 (59%)	600 (71%)	<0.001
MV, n (%)	3,058 (91%)	753 (89%)	768 (91%)	782 (93%)	755 (89%)	0.046
CRRT, n (%)	250 (7%)	16 (2%)	50 (6%)	85 (10%)	99 (12%)	<0.001
Los hospital (days)	9.99 (6.63, 16.01)	8.24 (5.68, 13.13)	9.85 (6.49, 15.67)	11.55 (7.03, 17.51)	11.72 (7.28, 17.84)	<0.001
Hospital Mortality, n (%)	451 (13%)	68 (8%)	97 (11%)	118 (14%)	168 (20%)	<0.001
Los ICU (days)	3.59 (2.34, 6.14)	3.05 (2.16, 4.22)	3.67 (2.42, 5.95)	4.18 (2.78, 7.39)	4.12 (2.43, 7.80)	<0.001
ICU Mortality, n (%)	317 (9%)	35 (4%)	68 (8%)	84 (10%)	130 (15%)	<0.001
28-day hospital Mortality, n (%)	535 (16%)	92 (11%)	117 (14%)	144 (17%)	182 (22%)	<0.001
90-day hospital Mortality, n (%)	785 (23%)	140 (17%)	170 (20%)	215 (25%)	260 (31%)	<0.001

SBP, Systolic blood pressure; DBP, Diastolic blood pressure; Spo_2_, oxygen saturation; SOFA, Sequential organ failure assessment; LODS, Logistic Organ Dysfunction System; CCI, Charlson Comorbidity Index; AKI, Acute kidney injury; RBC, Red blood cell count; WBC, White blood cell count; GV, Glycemic variability; BUN, Blood urea nitrogen; INR, International normalized ratio; PT, Prothrombin time; PPT, Partial thromboplastin time; MV, Mechanical Ventilation; CRRT, Continuous renal replacement therapy; LOS, Length of Stay.

### Baseline characteristics

The baseline features of critically ill patients with mitral valve disease were presented in **Table 1**. The median values of GV for each quartile were 9.57 (IQR: 6.74–11.52), 16.09 (IQR: 14.66–17.69), 23.07 (IQR: 20.84–25.33), and 36.94 (IQR: 31.89–45.82), respectively. Patients in the highest GV quartile exhibited a greater incidence of diabetes and sepsis, reported higher SBP, decreased DBP, increased CCI scores, as well as elevated levels of BUN and creatinine compared to those in the lower quartiles. In addition, individuals situated in the upper quartiles of GV had longer durations of hospital stays (8.24 days vs. 9.85 days vs. 11.55 days vs. 11.72 days, *P* < 0.001) and prolonged ICU admissions (3.05 days vs. 3.67 days vs. 4.18 days vs. 4.12 days, *P* < 0.001). Moreover, mortality rates grew significantly with rising GV, with the 28-day hospital mortality climbing from 11% to 22% and the 90-day hospital mortality increasing from 17% to 31% across the quartiles (*P* < 0.001).

### Primary outcomes

**Figure 3:**
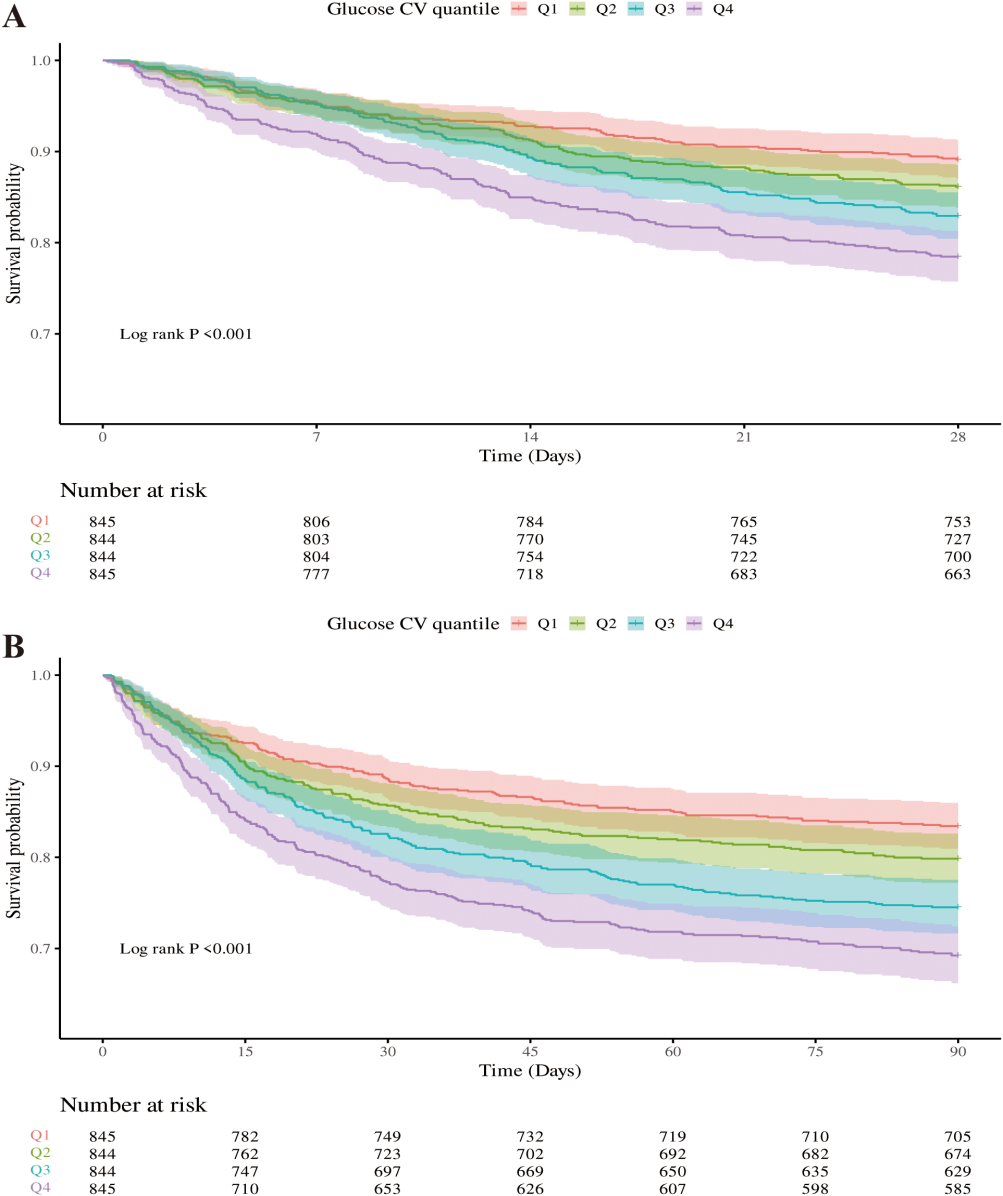
Kaplan-Meier survival curves were used to analyze the mortality rates of critically ill patients with mitral valve disease over two time points: 28 days **(A)** and 90 days **(B)**.

**Figure 3** showed that Kaplan-Meier survival curves were used to evaluate the occurrence of primary and secondary outcomes among groups divided by GV index quartiles. Patients with elevated GV demonstrated a significantly increased risk of 28-day (**Figure 3A**) and 90-day (**Figure 3B**) mortality (Log-rank *P* < 0.001). Univariable Cox regression results for 28-day all-cause mortality in critically ill patients with mitral valve disease were presented in **Supplementary Table S3**. Variables recognized as statistically significant (*P* < 0.05) during univariate analysis, together with clinically pertinent factors suggested by medical professionals relying on their clinical experience, were included as independent variables. An analysis using Cox proportional hazards was performed to explore the relationship between GV and mortality within 28 days. The results demonstrated that an increase in GV by 10 units was persistently linked to a higher risk of mortality in all examined models: the unadjusted model (Model 1) showed [HR: 1.14, 95% CI: 1.09 – 1.19, *P* < 0.01], the partially adjusted model (Model 2) showed [HR: 1.15, 95% CI: 1.10 – 1.20, *P* < 0.01], and the fully adjusted model showed (Model 3) [HR: 1.13, 95% CI: 1.08 – 1.18, *P* < 0.01]. Patients in the highest GV index quartile, when treated as a nominal variable, showed a significant association with an elevated risk of 28-day hospital death in the three Cox proportional hazards models: unadjusted model (model 1) [HR, 2.11 (95% CI 1.64–2.71) *P* < 0.01], partly adjusted model (model 2) [HR, 2.19 (95% CI 1.71–2.82) *P* < 0.01] and fully adjusted model (model 3) [HR, 2.00 (95% CI 1.53–2.61) *P* < 0.01], compared to subjects in the lowest quartile. Comparable results were identified in the multivariate Cox proportional hazards evaluation concerning GV and mortality after 90 days (**Table 2**).

**Table 2:** Association between GV and mortality.

Variables	Model 1		Model 2		Model 3	
	HR (95%CI)	*P*	HR (95%CI)	*P*	HR (95%CI)	*P*
D28						
Glucose CV10	1.14 (1.09 ~ 1.19)	<.001	1.15 (1.10 ~ 1.20)	<.001	1.13 (1.08 ~ 1.18)	<.001
Glucose CV quantile						
Q1	1.00 (Reference)		1.00 (Reference)		1.00 (Reference)	
Q2	1.29 (0.98 ~ 1.69)	0.068	1.28 (0.98 ~ 1.69)	0.075	1.33 (1.01 ~ 1.75)	0.045
Q3	1.60 (1.23 ~ 2.08)	<.001	1.60 (1.23 ~ 2.08)	<.001	1.63 (1.25 ~ 2.13)	<.001
Q4	2.11 (1.64 ~ 2.71)	<.001	2.19 (1.71 ~ 2.82)	<.001	2.00 (1.53 ~ 2.61)	<.001
D90						
Glucose CV10	1.13 (1.09 ~ 1.17)	<.001	1.13 (1.09 ~ 1.17)	<.001	1.11 (1.07 ~ 1.15)	<.001
Glucose CV quantile						
Q1	1.00 (Reference)		1.00 (Reference)		1.00 (Reference)	
Q2	1.24 (0.99 ~ 1.55)	0.058	1.23 (0.98 ~ 1.54)	0.073	1.27 (1.01 ~ 1.59)	0.042
Q3	1.61 (1.30 ~ 1.99)	<.001	1.57 (1.27 ~ 1.95)	<.001	1.59 (1.28 ~ 1.97)	<.001
Q4	2.04 (1.66 ~ 2.51)	<.001	2.08 (1.69 ~ 2.56)	<.001	1.85 (1.48 ~ 2.30)	<.001

HR, Hazard Ratio; CI, Confidence Interval.

Model1: Crude.

Model2: Adjust: Sex, Race, Insurance, Smoker, Age, Weight.

Model3: Adjust: Sex, Race, Insurance, Smoker, Congestive heart failure, Diabetes, Insulin, Age, Weight, SBP, Systolic blood pressure; CCI ,Charlson Comorbidity Index; Potassium, Creatinine; INR, International normalized ratio.

**Figure 4:**
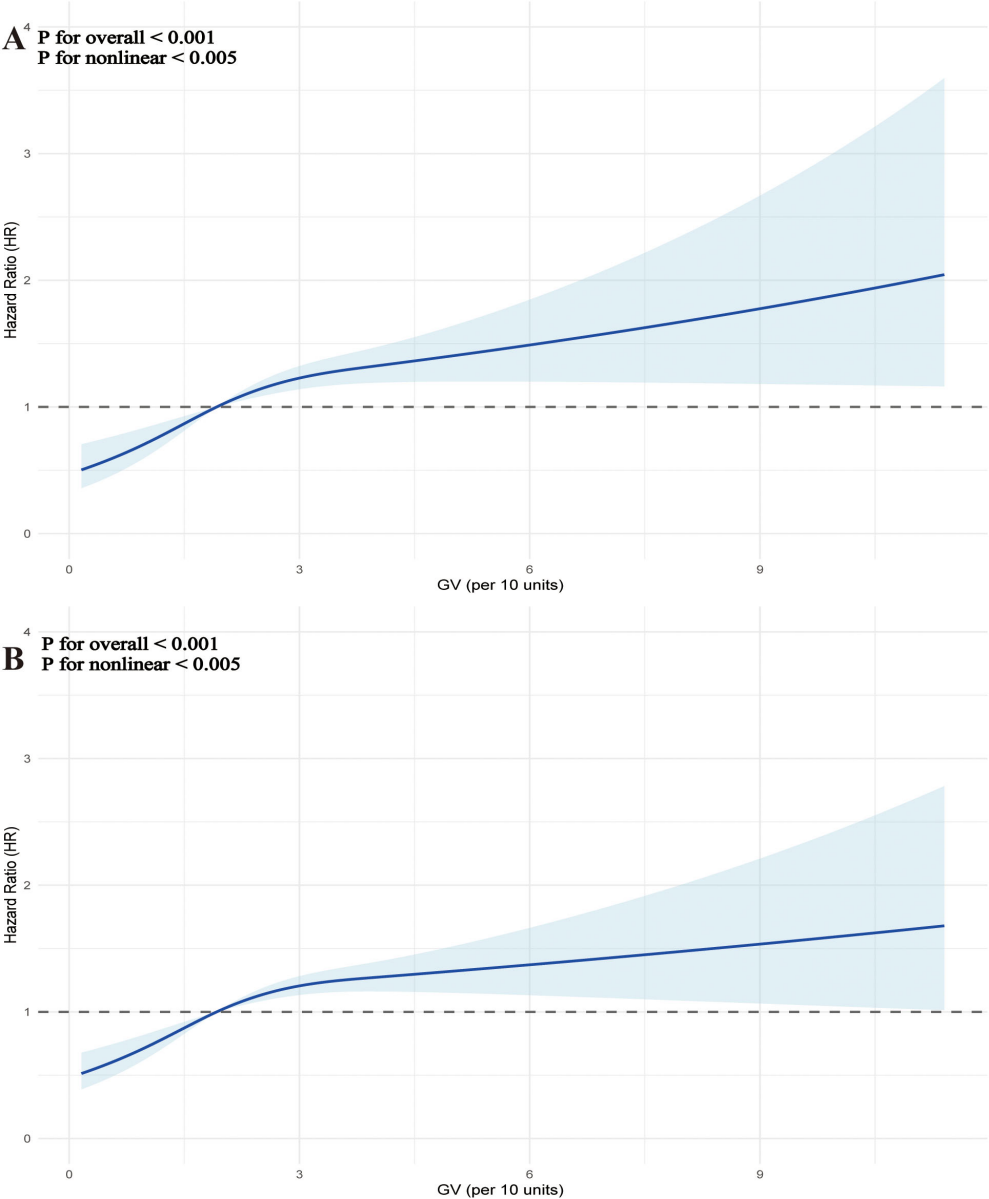
Restrictive cubic spline (RCS) curves were used to analyze the nonlinear relationship between glycemic variability (GV) and mortality in critically ill patients with mitral valve disease at two time points: 28 days **(A)** and 90 days **(B)**.

Additionally, the regression models using RCS, which accounted for covariates, demonstrated a non-linear relationship between increasing GV and the risk of mortality at both 28 days (**Figure 4A**) and 90 days (**Figure 4B**) (P for overall <0.001, P for non-linearity < 0.005 for 28-day mortality; P for overall <0.001, P for non-linearity <0.005 for 90-day mortality). **Table 3** summarizes the threshold effect analysis. For 28-day mortality, the inflection point of GV was 38.4. Below this threshold, GV was significantly associated with increased risk (HR = 1.26, 95% CI: 1.12–1.42, *P* <.001); above the threshold, the association was not significant (HR = 1.00, 95% CI: 0.89–1.12, *P* = 0.985). The likelihood ratio test indicated a significant threshold effect (*P* = 0.008). For 90-day mortality, the inflection point was 38.7. Below this value, GV was significantly associated with mortality (HR = 1.25, 95% CI: 1.14–1.37, *P* <.001); above it, no significant association was found (HR = 0.98, 95% CI: 0.89–1.09, *P* = 0.743). The likelihood ratio test remained significant (*P* <.001).

**Table 3:** Threshold effect analysis of GV on Mortality.

Outcome	Effect	*P*
D28		
GV/10		
Model 1 Fitting model by standard linear regression	1.13 (1.08 - 1.18)	<.001
Model 2 Fitting model by two-piecewise linear regression		
Inflection point	38.4	
<38.4	1.26 (1.12 - 1.42)	<.001
≥38.4	1.00 (0.89 - 1.12)	0.985
P for likelihood test		0.008
D90		
GV/10		
Model 1 Fitting model by standard linear regression	1.11 (1.07 - 1.15)	<.001
Model 2 Fitting model by two-piecewise linear regression		
Inflection point	38.7	
<38.7	1.25 (1.14 - 1.37)	<.001
≥38.7	0.98 (0.89 - 1.09)	0.743
P for likelihood test		<.001

### Subgroup analysis

To examine if the influence of GV on the primary outcome varied across different subgroups of the population, a predetermined subgroup analysis was conducted. The HRs and 95% CIs for each subgroup were assessed, along with interaction *P* values to evaluate possible modifications in effects.

**Figure 5:**
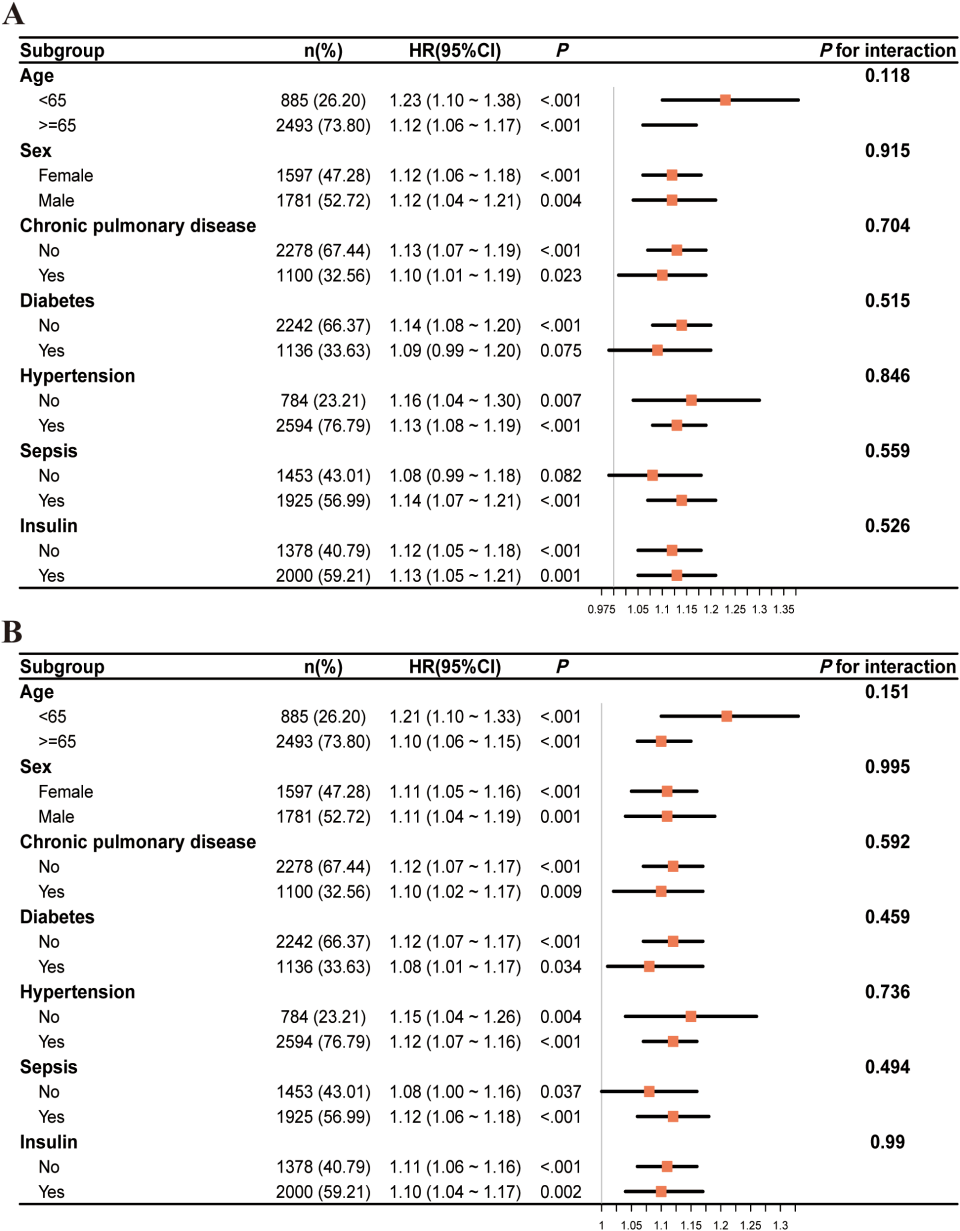
The subgroup analysis of critically ill patients with mitral valve disease at two time points: 28 days **(A)** and 90 days **(B)**.

Overall, HRs were consistently greater than 1 across all subgroups, indicating an increased risk, although the degree of statistical significance varied. Stratification by age, sex, chronic pulmonary disease, hypertension, sepsis, and insulin use showed consistent associations with no significant interaction effects (all interaction *P* values > 0.05). Among patients without diabetes, the HR was significantly elevated (1.14, 95% CI: 1.08–1.20, *P* < 0.001), whereas in those with diabetes, the HR (1.09, 95% CI: 0.99–1.20) was not statistically significant (*P* = 0.075); however, the interaction *P* value (0.515) indicated no significant effect modification (**Figure 5A**). Similar trends were observed in the subgroup analysis of secondary outcomes (**Figure 5B**).

### Sensitivity analyses

As shown in **Supplementary Table S4**, sensitivity analyses yielded consistent results. GV was significantly associated with 60-day mortality (HR = 1.12, 95% CI: 1.07–1.16, *P* <.001), 28-day mortality in non-diabetic patients (HR = 1.14, 95% CI: 1.08–1.20, *P* <.001), and 90-day mortality in non-diabetic patients (HR = 1.12, 95% CI: 1.07–1.17, *P* <.001).

## Discussion

The relationship between GV and clinical outcomes in severely ill patients suffering from mitral valve disease was examined using data obtained from a cohort in the United States. The results indicated a significant correlation between heightened GV and the increase in all-cause mortality rates at both 28 days and 90 days. Importantly, this association remained robust even after adjusting for potential confounding factors. Moreover, the RCS regression models revealed a nonlinear upward trend connecting GV with mortality rates at both 28 and 90 days. This suggested that as GV escalated, the risk of mortality did not increase in a straightforward linear manner but rather adhered to a more intricate, nonlinear progression. Additionally, the relationship between GV and mortality at both 28 and 90 days exhibited a comparable pattern, indicating that GV maintained a level of consistency in forecasting short-term outcomes, particularly in ICU patients experiencing significant physiological stress responses during the acute phase. This further emphasized the critical nature of managing GV (26, 27).

Physiologically, glucose metabolism was essential for cardiovascular health because the heart primarily used glucose as its energy source (28). The metabolic processing of glucose played a vital role in maintaining the physiological integrity of the cardiovascular system. Disruptions in this metabolic equilibrium, especially within affected heart tissues, served as significant catalysts for the onset and progression of cardiovascular diseases.

Numerous studies have established a link between GV and outcomes in ICU patients. For instance, Shen et al.’s prospective cohort study of 29,260 patients with type 2 diabetes found a significantly increased incidence of cardiovascular disease in patients with large fluctuations in HbA1c (29). While the study group mainly consisted of non-intensive care unit (ICU) patients, it indicated that prolonged GV might also have elevated the risk of cardiovascular issues. Furthermore, Egi et al. examined information from 7,021 ICU patients and discovered that elevated GV, which was assessed through the standard deviation of blood glucose levels, significantly correlated with a rise in 28-day mortality. Those categorized in the high GV group were confronted with roughly a 30% increased risk of mortality (30). Additionally, a different prospective cohort study involving 4,982 participants examined how fluctuations in fasting glucose levels affected cardiovascular incidents and mortality rates, indicating that higher variability in blood glucose correlated with a greater risk of mortality (31), thereby reinforcing the evidence connecting GV to adverse health outcomes.

As an important component of glycemic control, GV has received extensive attention in recent years. Compared with persistent hyperglycemia, GV showed more significant pathological effects in inducing oxidative stress, promoting inflammatory response, and impairing vascular endothelial function (14, 32). Monnier et al. demonstrated a significant relationship between acute blood glucose fluctuations and oxidative stress activation (14). Antonio Ceriello and colleagues have demonstrated that postprandial hyperglycemia triggered excessive production of superoxide anions. These anions reacted with nitric oxide to form peroxynitrite, a potent oxidant, leading to nitrosative stress and the formation of reactive nitrogen species such as nitrotyrosine. The cytotoxic effects of these compounds damaged endothelial cells, contributing to both microvascular and macrovascular complications associated with diabetes (33). Furthermore, GV was associated with increased platelet activity and elevated coagulation factors, which may have exacerbated cardiovascular events by enhancing thrombophilia (32). In patients with valvular heart disease, pathologic changes usually involve degeneration of valve structure, calcification, fibrosis, and secondary heart failure. Previous studies showed that abnormal glucose metabolism could accelerate the process of valve tissue remodeling (8). Through its combined effects on vascular endothelium, metabolism and immunity, GV was considered an important contributing factor for the prognosis of patients with valvular disease.

In subgroup analyses, GV consistently correlated with mortality across age, sex, and comorbidity groups. However, the association was attenuated in patients with diabetes (HR 1.09; 95% CI 0.99–1.20; *P* = 0.075), suggesting potential metabolic adaptation to glycemic fluctuations (13, 14). A similar phenomenon was reported in the study of chronic hyperglycemia by Brownlee et al., in which long-term hyperglycemic states may have reduced an individual’s stress response to acute blood glucose fluctuations (13). Firstly, Mitochondrial overgeneration of reactive oxygen species under chronic hyperglycemia sustained oxidative stress in diabetes (34). Hyperglycemia-induced advanced glycation end-products (AGEs) production and protein kinase C (PKC) activation redirected cellular damage away from acute oxidative injury toward chronic pathological pathways (34). These adaptations might have reduced the relative impact of acute GV-induced oxidative bursts (35). Consequently, blunted oxidative stress reactivity in patients with diabetes might have diminished glycemic variability’s impact on short-term mortality (14). Secondly, prolonged exposure to hyperglycemia impaired β-cell function but enhanced peripheral tissue adaptation to glucose fluctuations (36). Thirdly, skeletal muscle and adipose tissue insulin resistance in diabetes promoted alternative fuels (e.g., free fatty acids) use, decreased reliance on glucose uptake and buffered glycemic variability-induced injury (36). Lastly, Diabetic neuropathy dampened hypothalamic-pituitary-adrenal (HPA) axis and catecholamine responses to glucose shifts, limited stress adaptation and lessened the physiological strain of glycemic variability (37). Systemic adaptations might have explained the weakened GV-mortality association in diabetes, though further research is needed to clarify glucose metabolism’s influence on GV pathophysiology.

According to this study, GV appeared as a potential independent risk factor for critically ill individuals with mitral valve disease. Elevated GV levels were linked to increased mortality and adverse clinical outcomes in this population. Therefore, monitoring GV could have served as a valuable tool for clinicians in risk stratification and decision-making processes. Implementing strategies to manage GV may improve patient outcomes in the intensive care setting. GV levels should be monitored and controlled not only in the clinical management of patients in general, but also in critically ill patients in the ICU, especially for non-diabetic ICU patients. GV monitoring was often neglected in current clinical practice, and it was recommended that GV be included in the glycemic control strategy of critically ill patients, and intervention thresholds might have been set (26). Continuous glucose monitoring (CGM) can be considered to improve glycemic control, reduce both diabetes-related events and overall health management costs (27).

This study provided valuable insights into the relationship between mitral valve disease and GV, although several limitations merited consideration. The retrospective observational design introduced inherent biases and potential confounding, limiting causal interpretations. Owing to limitations of the MIMIC-IV database, key variables such as body mass index (BMI), waist circumference, alcohol use, baseline liver disease, medication use (e.g., insulin, antihypertensives, lipid-lowering agents), lifestyle, diet, the severity of mitral valve disease and socioeconomic status were unavailable or largely missing and could not be adjusted for. Although all patients had at least three glucose readings, variations in measurement frequency and timing may have affected GV estimates. In this study, GV was calculated following established methods from previous research (22). Exclusion of patients with fewer readings may have introduced selection bias towards more critically ill individuals. Furthermore, the absence of controls for nutritional interventions and glucose-modifying therapies might have influenced the results. The restriction to ICU populations and reliance on the MIMIC-IV database, covering an extended period of evolving clinical practice, limited the external validity of the findings. Additionally, as the cohort was drawn exclusively from the U.S., applicability to other settings remained uncertain. Importantly, the study assessed GV only during ICU admission, overlooking potential post-discharge variability that could have impacted long-term outcomes. Further prospective research is required to address these limitations.

## Conclusion

In the context of critically ill patients suffering from mitral valve disease, greater GV levels were significantly related to mortality from all causes at 28 and 90 days after ICU admission. GV levels are crucial for assessing mortality risk in mitral valve disease patients admitted to the ICU. Monitoring GV levels in mitral valve disease patients in the ICU could be a key factor in accurately observing patients and planning subsequent treatments. The evaluation and control of GV may become a new target to improve the prognosis of patients with severe valvular disease, which deserves great attention from clinicians and researchers.

## Data Availability

Publicly available datasets were analyzed in this study. This data can be found here: https://mimic.physionet.org.
