# Supplementary material for: A retrospective analysis of the prognostic implications of glycemic variability on all-cause mortality in critically ill patients with mitral valve disease
Source: Front Endocrinol (Lausanne). 2025 Jul 14;16:1620762. doi: 10.3389/fendo.2025.1620762 (PMC12301203; doi:10.3389/fendo.2025.1620762)
Supplement: Supplementary file 1 [file Table1.docx]

**Supplementary material**

**Table S1 Specific ICD codes used for mitral valve disease identification**

'I05','3942','09321','I052','I080','Q23','I3481','I340','I083','3949','3969','I3489','3962','3940','Q239','3961','3941','4240','I342','3968','I081','Q232','3960','I050','I348','7465','I34','I059','3963','7466','Q233','Q238','I051','I349','I341','I058'

**Table S2 Missing variables and their percentages**

| Column | Missing Percentage (%) |
| --- | --- |
| Weight | 11.5748964 |
| Temperature | 11.3084665 |
| PTT | 6.4239195 |
| INR | 6.0686797 |
| PT | 6.0686797 |
| Urine output | 2.9603316 |
| RBC | 0.3848431 |
| WBC | 0.3848431 |
| Platelet | 0.3552398 |
| Potassium | 0.2960332 |
| Creatinine | 0.2368265 |
| Sodium | 0.2072232 |
| BUN | 0.2072232 |
| Respiratory rate | 0.1776199 |
| Heart rate | 0.1480166 |
| SBP | 0.1480166 |
| DBP | 0.1480166 |
| Spo2 | 0.1480166 |
| SOFA | 0.1480166 |

PPT: Partial thromboplastin time, INR: International normalized ratio, PT: Prothrombin time, RBC: Red blood cell count, WBC: White blood cell count, BUN: Blood urea nitrogen, SBP: Systolic blood pressure, DBP: Diastolic blood pressure, Spo_2_: oxygen saturation, SOFA: Sequential organ failure assessment.

**Table S3 Univariable cox regression for 28-day all-cause mortality**

| **Variables** | ***P*** | **HR (95%CI)** |
| --- | --- | --- |
|  |  |  |
| Sex |  |  |
| Female |  | 1.00 (Reference) |
| Male | 0.517 | 0.95 (0.80 ~ 1.12) |
| Race |  |  |
| Black |  | 1.00 (Reference) |
| Other | 0.821 | 1.04 (0.73~1.48) |
| White | 0.762 | 0.95 (0.70 ~1.30) |
| Age | **<.001** | 1.04 (1.03 ~ 1.05) |
| Weight | 0.379 | 1.00 (0.99 ~ 1.00) |
| SBP | **0.002** | 0.99 (0.98 ~ 0.99) |
| DBP | 0.992 | 1.00 (0.99 ~ 1.01) |
| SOFA | 0.088 | 1.03 (1.00 ~ 1.06) |
| LODS | **<.001** | 1.23 (1.20 ~ 1.26) |
| CCI | **<.001** | 1.22 (1.18 ~ 1.25) |
| Congestive heart failure |  |  |
| No |  | 1.00 (Reference) |
| Yes | **<.001** | 1.59 (1.30 ~ 1.96) |
| Diabetes |  |  |
| No |  | 1.00 (Reference) |
| Yes | **0.042** | 1.20 (1.01 ~ 1.43) |
| Hypertension |  |  |
| No |  | 1.00 (Reference) |
| Yes | 0.210 | 1.14 (0.93 ~ 1.41) |
| AKI |  |  |
| No |  | 1.00 (Reference) |
| Yes | **<.001** | 3.31 (2.20 ~ 4.98) |
| Sepsis |  |  |
| No |  | 1.00 (Reference) |
| Yes | **<.001** | 2.58 (2.11 ~ 3.14) |
| Urine output | **<.001** | 0.99 (0.99 ~ 0.99) |
| Sodium | 0.270 | 0.99 (0.97 ~ 1.01) |
| Potassium | **0.015** | 1.20 (1.04 ~ 1.39) |
| BUN | **<.001** | 1.02 (1.01 ~ 1.02) |
| Creatinine | **<.001** | 1.13 (1.10 ~ 1.17) |
| INR | **<.001** | 1.22 (1.17 ~ 1.27) |
| PT | **<.001** | 1.02 (1.02 ~ 1.03) |
| PTT | **<.001** | 1.01 (1.01 ~ 1.01) |
| Norepinephrine |  |  |
| No |  | 1.00 (Reference) |
| Yes | **<.001** | 2.56 (2.16 ~ 3.03) |
| Neuroblock |  |  |
| No |  | 1.00 (Reference) |
| Yes | **<.001** | 3.67 (2.65 ~ 5.08) |
| Insulin |  |  |
| No |  | 1.00 (Reference) |
| Yes | **<.001** | 0.57 (0.48 ~ 0.68) |
| MV |  |  |
| No |  | 1.00 (Reference) |
| Yes | 0.854 | 1.03 (0.77 ~ 1.38) |
| CRRT |  |  |
| No |  | 1.00 (Reference) |
| Yes | **<.001** | 2.74 (2.18 ~ 3.45) |

SBP: Systolic blood pressure, DBP: Diastolic blood pressure, SOFA: Sequential organ failure assessment, LODS: Logistic Organ Dysfunction System, CCI: Charlson Comorbidity Index, AKI: Acute kidney injury, BUN: Blood urea nitrogen, INR: International normalized ratio, PT: Prothrombin time, PPT: Partial thromboplastin time, MV: Mechanical Ventilation, CRRT: Continuous renal replacement therapy.

**Table S4 Sensitivity analysis for the association between GV and Mortality**

| **Variables** | ***P*** | **HR (95%CI)** |
| --- | --- | --- |
| 60-Day Mortality | **<.001** | 1.12 (1.07 ~ 1.16) |
| 28-Day Mortality in Non-Diabetic Patients | **<.001** | 1.14 (1.08 ~ 1.20) |
| 90-Day Mortality in Non-Diabetic Patients | **<.001** | 1.12 (1.07 ~ 1.17) |
